# Phoneme-cued coaching during standard earpopper^®^ use

**DOI:** 10.1007/s00405-026-10117-y

**Published:** 2026-03-09

**Authors:** Bulent Mamikoglu, Kristina Piastro, Robert Ward

**Affiliations:** 1 Department of Otolaryngology Head and Neck Surgery, Westchester Medical Center, Valhalla, NY USA; 2 Department of Otolaryngology—Head and Neck Surgery, New York Medical College, Valhalla, NY USA; 3 Department of Neurosurgery, College of Medicine Peoria, University of Illinois, Peoria, IL USA

**Keywords:** Eustachian tube dysfunction, Otitis media with effusion, EarPopper®, Autoinflation, Politzerization, Patient instruction

## Abstract

This technical note describes a standardized coaching method that integrates a simple phoneme-based cue into standard EarPopper^®^ use, without altering device settings, airflow, or dosing. The technique is intended to facilitate coordination between swallowing and soft-palate elevation during positive nasopharyngeal insufflation, a prerequisite for Eustachian tube (ET) opening. Explicit procedural guidance is provided, including patient selection, contraindications, step-by-step technique, the number of insufflations–swallow cycles per session, clinic onboarding, a clinic-first followed by home-use protocol, troubleshooting, and suggested monitoring tools. This article does not present outcome data or claim clinical efficacy; the technique is proposed as a behavioral coaching adjunct consistent with established concepts of ET physiology and fully compliant with the manufacturer’s Instructions for Use. Clinical effectiveness has not been formally tested in a comparative study; the technique is presented as a proposed coaching adjunct to standard device use.

**Supplementary Information:**

The online version contains supplementary material available at 10.1007/s00405-026-10117-y.

## Introduction and rationale

Effective eustachian tube opening depends on coordinated activation of the tensor and levator veli palatini muscles during swallowing, allowing transient aeration of the middle ear. This physiologic principle has been recognized since early descriptions of tubal mechanics and autoinflation maneuvers, including Politzerization and its later refinements [1].

Classic otologic texts and modern pediatric otology literature emphasize that impaired or inconsistent tubal opening contributes to middle-ear pressure dysregulation and otitis media with effusion (OME) [2]. Contemporary diagnostic approaches, including endoscopic visualization, further demonstrate that successful ET opening is a dynamic, swallow-dependent event rather than a passive pressure phenomenon [3].

Standard EarPopper^®^ use has been evaluated in selected clinical contexts [4]; however, consistent execution can be limited by difficulty timing a swallow during airflow. The present manuscript describes a coaching method intended to may help instructional consistency and patient execution, without presenting outcomes.

Production of a voiceless velar plosive (/k/) requires posterior tongue dorsum elevation and transient contact with the soft palate. This maneuver recruits coordinated activity of the styloglossus and intrinsic tongue musculature, followed by activation of the soft palate through the levator veli palatini and tensor veli palatini muscles—closely mirroring the neuromuscular sequence required for physiologic ET opening during swallowing [3, 5–7]. From a neuroanatomic perspective, tongue dorsum elevation is mediated primarily by the hypoglossal nerve (CN XII), while soft-palate elevation involves the pharyngeal plexus (predominantly CN X). Critically, the tensor veli palatini—the principal active opener of the ET—is innervated by the mandibular division of the trigeminal nerve (CN V3), providing a fast and reproducible somatic motor pathway [5, 6].

The present manuscript describes a coaching strategy that uses a “K” posture immediately followed by a swallow to prime this neuromuscular coordination. The vowel component of the syllable “KA” does not contribute directly to ET opening but serves to maintain a posterior oral cavity configuration and reduce jaw or laryngeal tension, making the maneuver comfortable and reproducible. The technique relies on neuromuscular priming rather than pressure generation and does not involve Valsalva-like effort.

### Indications and patient selection

This coaching technique is intended primarily for cooperative adolescents and adults using EarPopper^®^ for clinically appropriate autoinflation in the setting of suspected obstructive ET dysfunction or baro-challenge symptoms. Use in younger children may be limited by the ability to maintain nasal seal, follow multi-step instructions, and initiate a deliberate swallow on cue; therefore, pediatric application should be individualized and generally reserved for children who can reliably perform the sequence under supervision. Cooperation and cognitive capacity are prerequisites for safe home use. Additional user instructions for the EarPopper is available through manufactures websites [8].

This coaching technique may be considered in cooperative adolescents and adults with:

- Obstructive Eustachian tube dysfunction presenting with aural fullness or pressure-equalization difficulty.
- Otitis media with effusion when autoinflation is clinically appropriate, in the presence of no active nasal purulent drainage [2, 4].
- Baro-challenge symptoms without evidence of fixed anatomic obstruction.

Patients must be able to follow verbal instructions and reliably initiate a deliberate swallow on cue. Cognitive ability, cooperation, and comfort with the procedure are essential prerequisites. Application in younger children may be limited by these factors and should be individualized. The approach may be particularly relevant in patients with subjective ear fullness and minimal objective findings, where coordinated ET function is suspected rather than structural obstruction [9].

### Contraindications and cautions

Consistent with manufacturer guidance and standard otologic practice, EarPopper^®^ should not be used in patients with:

- Acute otitis media
- Upper respiratory infection or significant nasal congestion
- Active cold symptoms
- Known or suspected tympanic membrane perforation or presence of pseudomembrane prone to perforation.

The device is intended for single-patient use only. Treatment should be discontinued if pain, epistaxis, vertigo, or other concerning symptoms occur. We will repeat these in the upcoming sections.

### Equipment

- EarPopper^®^ EP-2100 A or EP-2100 device with single-patient nosepiece.
- Upright chair with head support.
- Water to assist swallowing if needed.
- Optional: flexible nasopharyngoscopy for instructional demonstration only [4].

### Definitions

- Insufflation–swallow cycle: one episode of nasopharyngeal airflow coordinated with one swallow.
- Pass: one cycle performed in each nostril (two cycles total).
- Treatment session (single use): two passes separated by a two to three-minutes interval (four total cycles).

### Step-by-step technique

1. Baseline assessment
  Perform otoscopy, otomicroscopy, additionally tympanometry, and symptom assessment (e.g., ETDQ-7) can be considered. Confirm absence of contraindications.
2. Positioning and seal
  Seat the patient upright. Insert the nosepiece into one nostril and occlude the contralateral nostril to ensure an airtight seal.
3. Swallow rehearsal
  Practice two to three dry swallows or swallows with water.
4. Phoneme cue, this the coaching
  Instruct the patient to assume a **silent velar “K” posture** (tongue dorsum elevated toward the soft palate), immediately followed by a normal swallow. Repeat with “KA”, “KAH” or “KAKKO” which is usually easy for the patient to do. (Fig. 1)

**Fig. 1:**
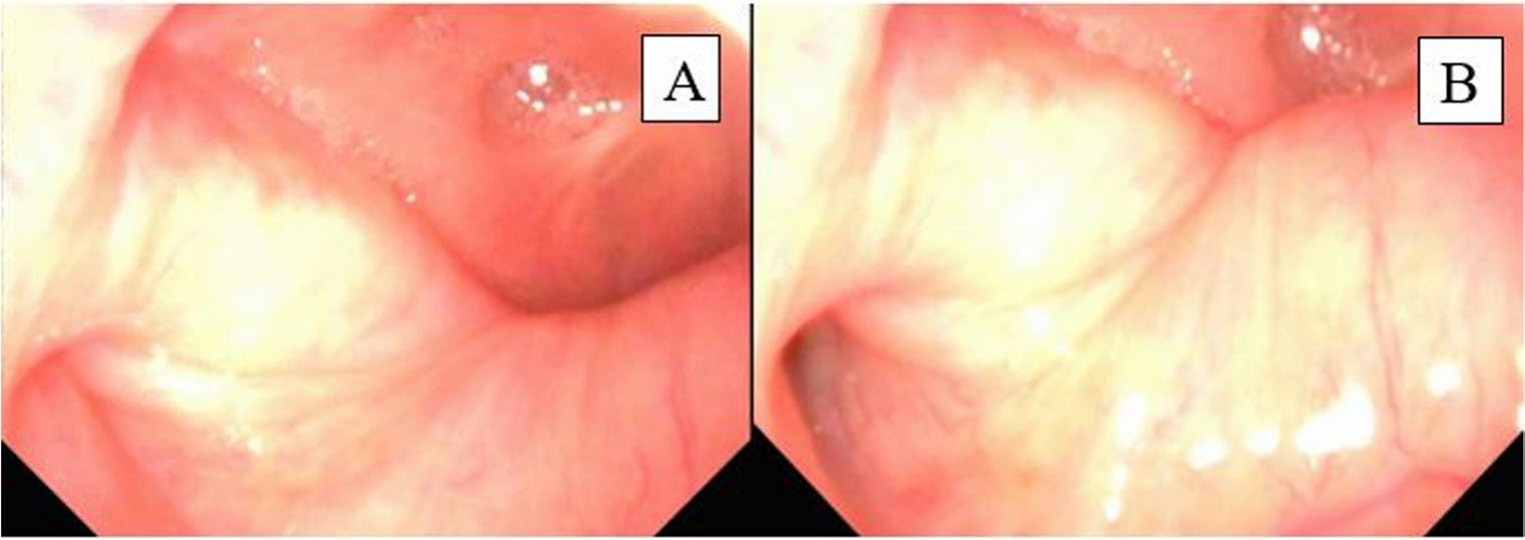
Endoscopic view of the nasopharyngeal orifice of the Eustachian tube. **A**. Resting (natural) position of the Eustachian tube orifice, demonstrating the collapsed configuration of the pharyngeal mucosa and minimal luminal opening. **B**. Active phonation of the word “kakko,” showing marked medial displacement and contraction of the surrounding soft palate musculature, consistent with activation of the tensor and levator veli palatini muscles. This maneuver enhances Eustachian tube patency and luminal dilation during velar plosive articulation
5. Pass 1
  Activate airflow and cue the patient to perform one swallow during airflow. Repeat in the opposite nostril
6. Inter-pass interval
  Wait two to three minutes
7. Pass 2
  Repeat the sequence in both nostrils.
  This completes one treatment session, consisting of four total insufflation–swallow cycles.
  The session can be repeated up to two to three times.

### Clinic vs. home use and dosing

The initial treatment session should be performed in the clinic under supervision to verify patient selection, nasal seal, timing of swallow during airflow, and tolerance. Once correct technique is demonstrated, subsequent sessions may be performed at home in appropriately selected patients, consistent with published EarPopper protocols [4].

### Dosing

- Per session: four total insufflation–swallow cycles.
- Frequency: two sessions daily (morning and evening) [4].
- Duration: typically, 2–4 weeks, depending on indication and tolerance.

Treatment should be suspended during upper respiratory infections or if otalgia develops.

### Home program

- Frequency: 2 sessions/day (AM and PM), unless IFU or clinician plan differs.
- Each session:
  - 1 pass per nostril → wait 2–3 min → repeat both nostrils (total 2 passes per nostril).
- Duration:
  - Published regimens vary; we generally recommend a several-week trial, individualized to symptoms and clinician guidance, and consistent with IFU.

### Follow-up and monitoring considerations

We follow up our patients in 4 to 6 weeks with otoscopic or otomicroscopic examination. The following measures are provided as suggested monitoring tools that clinicians may choose to use in routine care; they are not reported study outcomes in this technical note. Depending on clinician preference and availability, follow-up may include interval otoscopy/otomicroscopy, tympanometry, and symptom assessment (e.g., ETDQ-7). Maneuvers such as Valsalva/Toynbee may be used as part of routine functional assessment where appropriate. Persistence of symptoms despite appropriate technique should prompt reconsideration of diagnosis and management strategy.

### Complications and management

In routine clinical instruction of this maneuver, serious adverse events have not been encountered; however, patients must be counseled regarding potential risks and clear stop criteria.

- Otalgia or transient dizziness: pause treatment; reassess technique and nasal seal.
- Epistaxis or nasal discomfort: discontinue and evaluate nasal pathology before resuming.
- Severe otalgia or pain that does not resolve promptly.
- Sudden hearing change, new tinnitus spike with hearing drop, or aural bleeding.
- Persistent vertigo, new neurologic symptoms, or inability to ambulate safely.
- Otorrhea, especially purulent or bloody.
- Significant epistaxis or recurrent nasal bleeding.
- Any symptom that feels “wrong” compared with prior sessions.
- Suspected barotrauma: stop treatment and perform otologic evaluation. Patients should stop immediately and contact the treating clinician (or urgent care/ER depending on severity) for any of the following.

Failure to improve after 6–8 weeks should prompt reconsideration change the management strategy.

### Practical pearls

- The phoneme cue is a behavioral coaching aid, not a modification of the device.
- Proper nasal seal and swallow timing are more important than forceful effort.
- In-clinic demonstration may help patient confidence and adherence.
- Adherence to established dosing recommendations is essential.

## Supplementary Information

Below is the link to the electronic supplementary material.

Supplementary File 1. Video 1 (supplement). Demonstration of the phoneme-cued sequence (nasal seal followed by airflow then silent “K” posture and swallow). All visual material is fully anonymized and intended solely for instructional demonstration; no patient-specific data or outcomes are shown (PNG 457 KB)

## Data Availability

Not applicable (no new data were created or analyzed in this study).

## Declarations

### Language models use

During the preparation of this manuscript, the author utilized ChatGPT (OpenAI, GPT-4) solely as a formatting and editorial assistant — to align the article with journal-specific structure, style, and reference formatting requirements. The core content, clinical idea, scientific reasoning, and all written material were independently conceived, authored, and reviewed by Drs. Bulent Mamikoglu, Kristina Piastro and Robert Ward. No text was generated by AI that constitutes substantive scientific content or original analysis. ChatGPT was not used to create, interpret, or draft any portion of the research design or clinical claims, and was not involved in any authorship role.
