# Supplementary figures and images for: Phoneme-cued coaching during standard earpopper® use
Source: Eur Arch Otorhinolaryngol. 2026 Mar 9;283(6):4047–51. doi: 10.1007/s00405-026-10117-y (PMC13249776; doi:10.1007/s00405-026-10117-y)

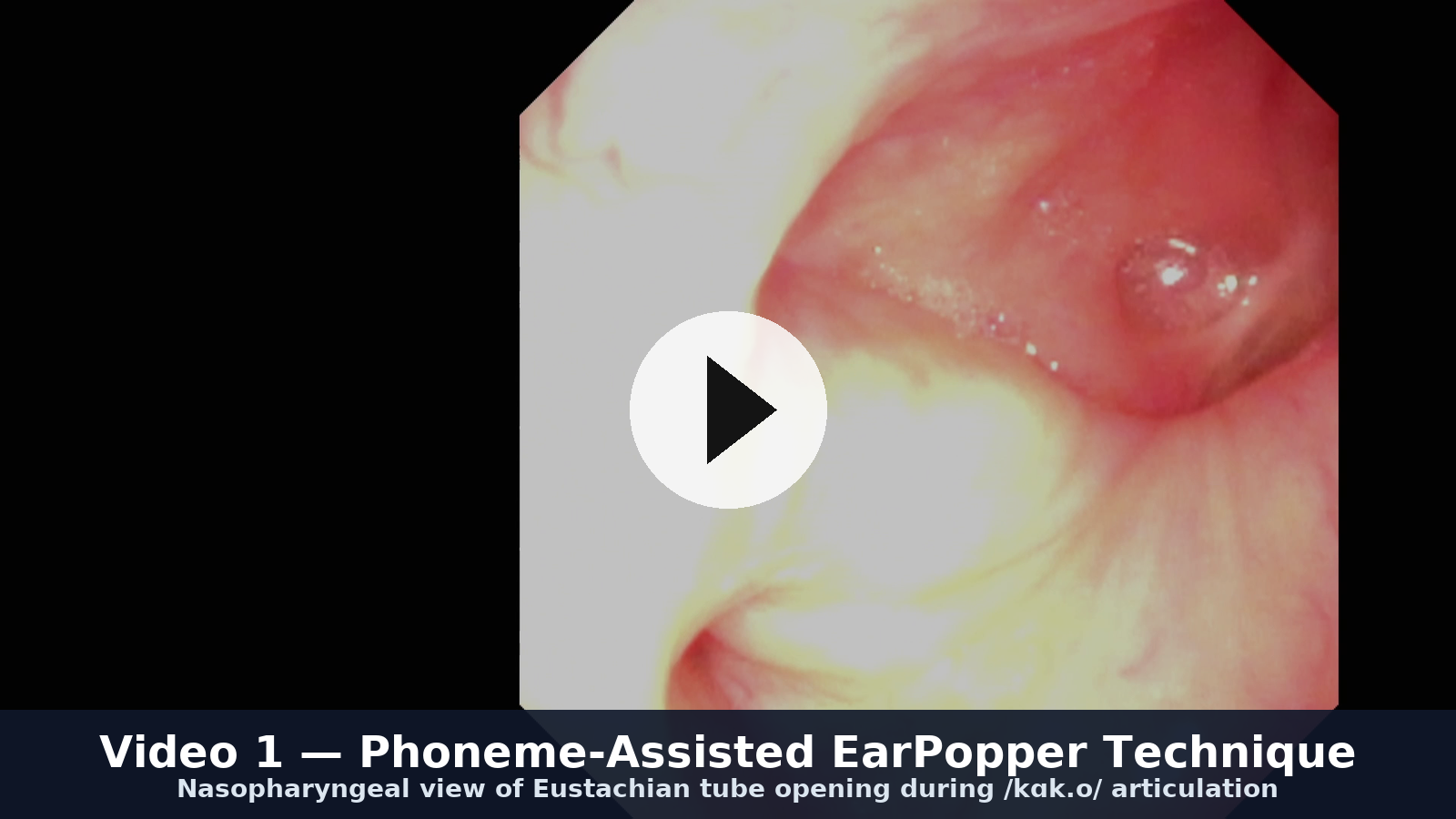

Supplement: Supplementary file 1 — Video 1 (supplement). Demonstration of the phoneme-cued sequence (nasal seal followed by airflow then silent “K” posture and swallow). All visual material is fully anonymized and intended solely for instructional demonstration; no patient-specific data or outcomes are shown (PNG 457 KB) [file 405_2026_10117_MOESM1_ESM.png]
